# Pelleted Sulla Forage (*Hedysarum coronarium* L.) as a Resource for Sheep Feeding Systems: In Vitro Nutritional Value and Sustainability Perspectives

**DOI:** 10.3390/ani15152322

**Published:** 2025-08-07

**Authors:** Barbara Piccirillo, Marialetizia Ponte, Marianna Pipi, Antonino Di Grigoli, Adriana Bonanno, Monica I. Cutrignelli, Alessandro Vastolo, Serena Calabrò

**Affiliations:** 1 Department of Veterinary Medicine and Animal Production, University of Naples Federico II, 80137 Napoli, Italy; monica.cutrignelli@unina.it (M.I.C.); alessandro.vastolo@unina.it (A.V.); serena.calabro@unina.it (S.C.); 2 Department of Agricultural, Food and Forestry Sciences, University of Palermo, 90128 Palermo, Italy; marialetizia.ponte@unipa.it (M.P.); marianna.pipi@unipa.it (M.P.); antonino.digrigoli@unipa.it (A.D.G.); adriana.bonanno@unipa.it (A.B.)

**Keywords:** forage preservation, secondary metabolites, polyphenols, condensed tannins, in vitro fermentation, methane mitigation, ruminant nutrition, volatile fatty acids

## Abstract

Enhancing forage protein is key to sustainable ruminant nutrition. The nutritive value of *Hedysarum coronarium* L. was investigated by studying different preservation systems (fresh vs. dehydrated pellet vs. hay) (Exp. 1) and morphological fractions (flowers vs. leaves vs. stems) (Exp. 2). For the fresh and pelleted systems, two cuts were used. Proximately, total polyphenols and condensed tannins were detected. In vitro fermentation characteristics were studied by incubating samples with buffered sheep rumen fluid, estimating methane production by volatile fatty acids. Fresh and pelleted sulla were more nutritionally advantageous than hay, in terms of metabolizable energy and protein-to-fiber ratio. Pelleting at the beginning of flowering proved to be a suitable forage for sheep feeding due to protein (15.1% DM), metabolizable energy (9.64 MJ/kg DM), structural carbohydrates (39.5% DM), and total polyphenols (13.5 GAE g/kg DM) content. This cut showed an in vitro fermentation rate (9.86 mL/h), organic matter degradability (55.7%), and volatile fatty acids (87.3 mmoL/g) that were higher (*p* < 0.05) than hay. Flowers and leaves showed higher (*p* < 0.05) levels of secondary metabolites than stems as well as lower methane production. These results suggest the potential influence of these compounds in reducing rumen emissions. Dehydration and pelleting resulted in an effective preservation method for maintaining nutrients in sulla forage.

## Simple Summary

Leguminous forages are important in sheep diets to ensure sufficient protein intake, valorize local resources, and enhance environmental sustainability. *Hedysarum coronarium* L. stands out for its nutritional qualities and polyphenols content, mainly consisting of condensed tannins. These beneficial metabolites improve the quality of animal products by increasing milk casein synthesis and influence rumen microbiota reducing methane production. Pelleting as a preservation method offers a valid option to ensure forage availability throughout the year. This in vitro study evaluated two cuts of sulla forage collected as fresh and preserved as dehydrated pellet and hay, and, for the fresh cuts, various parts of the plant (whole plant, flowers, leaves, and stems) were analyzed. Sulla forage harvested at the beginning of flowering and preserved as pellet is nutritionally valuable and suitable for sheep diets considering its crude protein (15.1% DM), metabolizable energy (9.64 MJ/kg DM), total polyphenols (13.57 GAE g/kg DM), in vitro fermentation rate (9.86 mL/h), organic matter degradability (55.7%), and volatile fatty acids production (87.3 mmoL/g). The condensed tannins and total polyphenols content in leaves and stems contribute to reduce methane emissions. Pelleting compared with haymaking resulted in an effective preservation method for maintaining nutrients in sulla and supports sustainable small ruminant nutrition.

## 1. Introduction

Sulla (*Hedysarum coronarium* L.), a biennial Leguminosae, provides high quality forage used for sheep nutrition whether directly grazed or preserved as hay. This species, native to Mediterranean countries, is widespread in parts of western North America and has been attracting interest in non-traditional areas (Australia and New Zealand) [1] due to its excellent adaptability to marginal environments and severe droughts, its versatility as a high-protein plant (20.2 ± 3.1% DM), and, more recently, its levels of phenolic compounds. 

Sulla is also believed to be a pioneer species in poor, compact, and degraded soils and, as a nitrogenous legume, can improve soil fertility for the next crop [2]. In 2023, it was cultivated on 97.694 hectares in Italy, accounting for 3.8% of the total area dedicated to temporary or alternate forages, which represents about 14% of the area planted with alfalfa [3]. Sulla is a predominantly erect plant with thick stems, generally hollow, which lignify rapidly after flowering. It has a robust and deep taproot with numerous secondary roots that penetrate clayey soils. The leaves are odd-pinnate, rather fleshy, and of variable shape, mostly oval. The inflorescences are axillary conical racemes with globose flowers ranging from light pink to bright red, to which are attributed hypocholesterolemic and laxative properties [4]. Sowing in monophyte can take place in autumn in areas with mild and rainy winters, in spring in colder climates, and in summer with reseeding in cereal crops and bare seed. The plant produces segmented legumes of brown color with a rough and spiny surface. Sulla has low water requirements and therefore thrives well in semi-arid and arid regions [5] but it is sensitive to cold [1]. It improves agricultural systems by adding nitrogen and organic matter to the soil and reducing erosion. It prefers well-drained, fertile, medium–fine, and calcareous soils and has a low tolerance to waterlogging and none to salinity. Spring utilization occurs through mowing and subsequent haymaking, while summer and autumn regrowths are suitable for grazing, which is also possible at the end of winter, allowing for the postponement of mowing and obtaining leafier forage. In the year of establishment, it should be lightly grazed (it is recommended to leave at least 10 cm of minimum height) to ensure good root development and enough plants for the second year. Mowing is primarily carried out after the first year since yield may not be optimal, and it should be done at the onset of flowering. Later, the stems may become woody and thus of lower quality, even though they may yield more. Mowing should be 20–30 cm above ground level to avoid collecting soil [6]. During haymaking, conditioning and raking can influence the success of hay due to leaf loss and thus protein loss. 

In addition to conservation systems, various other factors influence the nutritional quality of forage: species, variety, botanical family, plant developmental stage, cultivation methods, leaf/stem ratio, climatic conditions (temperature and rainfall), soils, and treatments. Sulla forage is highly palatable, due to the ratio of structural to non-structural carbohydrates [7] and it is characterized by secondary metabolites, as phenolic compounds (i.e., tannins) are mainly found in the leaf blades and flowers, especially during summer harvests [8]. The distribution of these compounds in tissues and cells also depends on their solubility: insoluble phenols are found in cell walls, while soluble ones are predominantly in vacuoles [9]. Moreover, heat stress characterized by high environmental temperatures could increase the concentration of polyphenols in different plant parts [10]. In monocotyledonous plants, such as grasses, tannins are not very prevalent, in contrast to dicotyledon families, particularly legumes, where they are in low to moderate concentrations (from 30–40 to 70 g/kg DM). 

While tannins are advantageous for plants, they have often been described negatively concerning animal production [11]. However, recent studies have shown a positive influence on ruminal microbiota and methane emissions. In particular, total polyphenols and total tannins present in diets containing hazelnuts and grape by-products significantly (*p* < 0.01) reduced the methane production related to either degraded OM or incubated OM [12]. Sulla forage grown in Mediterranean environments showed a non-constant tannin content (ranging from 8 to 50 g/kg DM) varying in function of the territory, specific part of the plant, phenological stage, and genotype [13,14]. At these concentrations, they do not cause harmful effects on intake, digestibility, or animal productivity; rather, they can (particularly proanthocyanidins) bind to proteins and reduce ruminal proteolysis [11,15], enhancing bypass proteins that are then digested and absorbed in the intestinal tract, thus improving the utilization of dietary proteins [16] and the synthesis of milk casein in mammary tissue [17]. In the blood of sheep receiving plant extracts rich in polyphenols through direct ruminal infusion, epicatechins (complex polyphenols) were found, indicating that metabolism had occurred [18,19]. A positive effect on environmental sustainability has also been observed, reducing in vitro methane emissions and nitrogen excretion [15], as well as reduced gastrointestinal nematode burden, due to these compounds’ ability to inhibit egg hatching and larval motility [20]. Some plant secondary metabolites (e.g., flavonoids and anthraquinoids) have the potential to decrease in vitro CH_4_ production in the rumen to 25% [21]. In sheep grazing on sulla, a more uniform distribution of feeding activity throughout the day was recorded, resulting in smaller, more frequent meals, which implies slower transit in the digestive tract. This promotes acetate formation and microbial protein synthesis, precursors of milk fats and casein, as well as slower passage through the small intestine, improving digestibility [17]. 

Forage preservation systems, both dehydration and haymaking, can influence the content, structure, and bioavailability of these compounds; in particular, thermal treatments seem to reduce phenolic components while improving their bioavailability [22]. Consequently, to exploit and stabilize such molecules in forage during periods of low availability, it is important to implement and research appropriate preservation techniques. The main objective of this investigation is to characterize sulla plants preserved by dehydration and pelleting. In particular, the influence of the preservation systems (fresh vs. pelleted vs. hay) (Exp. 1) and the effect of the different plant parts (whole plant vs. flowers vs. leaves vs. stems) (Exp. 2) were studied. The hypothesis is that pelleted sulla preserves its nutritional characteristics better than hay, moreover the secondary metabolites influence in vitro rumen fermentations, with a beneficial effect on the environment. 

## 2. Materials and Methods

### 2.1. Experimental Design

The investigation involved thirteen (No. 13) sulla (*Hedysarum coronarium* L.) forage samples collected in the south of Italy area (Sicily region, Agrigento province, 37°45′ N 13°36′ E, 670 m above sea level). In particular, the samples of sulla belong to the variety Landrace Avorio in the second year of cropping. Exp. 1: all plants of two fresh sulla forage amounts, each of about 400 kg, were harvested in the field, using a mower–shredder–loader machine, at the end of April and after two weeks, corresponding to two different phenological stages: beginning of flowering (1st cut, S1C_F) and full flowering (2nd cut, S2C_F). At both stages, after collecting representative fresh samples (each about 4 kg), the entire forage mass was immediately dehydrated in a small pilot system, consisting of a container with a capacity of about 400 kg per drying cycle, into which air, generated by a diesel-powered system, was blown at a speed of 3 m/s and a temperature below 55 °C. The chopped forage mass was placed on mesh platforms mounted on trolleys, which were then introduced into the container; here, the mass, exposed to hot air, remained at a temperature not exceeding 50 °C for about 18–24 h until a moisture level of about 15%, measured by a portable hygrometer based on electric conductibility, was reached. The dried forage for both cuts was then ground, passed through a small pelletizer in the facility to obtain pressed pellets (2.5 mm height, 0.6 mm diameter) which were packaged in separate bags (1st cut pelleted, S1C_P and 2nd cut pelleted, S2C_P). A representative sample of the fresh harvested sulla forage at full flowering (about 10 kg) was preserved by haymaking in accordance with local procedures (S_H). Exp. 2: fresh samples of whole plants (each about 6 kg) were collected in the field at two different times (beginning of flowering 1st cut, S1C_F, and full flowering 2nd cut, S2C_F) and were separated into different botanical parts (whole plant, flowers, leaves, and stems), before freeze-drying for analysis. All samples, which were ground using a 1 mm sieve (Brabender Wiley mill, Brabender OHG, Duisburg, Germany), were characterized by chemical composition, secondary metabolite content, and in vitro fermentation. Thus, the investigation involved the following two experiments: Exp. 1 with the aim of studying the influence of the preservation systems (fresh vs. pelleted vs. hay), where 5 sulla samples (2 fresh cuts, 2 pelleted, and 1 hay) were analyzed for chemical composition, secondary compound content, and incubated to study fermentation characteristics for 120 h; Exp. 2 with the aim to study the influence of methane production and secondary compound where 8 sulla samples (4 plant parts in 2 fresh cuts) were analyzed for chemical composition, secondary compound content, and incubated to study fermentation characteristics for 24 h including methane estimation.

### 2.2. Chemical Composition

The forage samples were analyzed in duplicate according to the official procedures of the Association of Official Analytical Chemists [23] to determine dry matter (DM, ID 934.01), ether extract (EE, ID 920.39), crude protein (CP, ID 2001.11), and ashes (ID 942.05). The fractions of structural carbohydrates, such as neutral detergent fiber (NDF), acid detergent fiber (ADF), and acid detergent lignin (ADL), were determined in accordance with Van Soest et al. [24] and expressed excluding residual ashes.

### 2.3. Determination of Condensed Tannins and Total Polyphenols

The determination of condensed tannins and total polyphenols was conducted on the extracts of all freeze-dried forage samples (0.75 g) in two replications. Extracts were prepared in duplicate as described by Gannuscio et al. [17]. They were then mixed with 25 mL of an acetone/water mixture (70:30, *v*/*v*), sonicated in a water bath with ultrasound (LBS1 Sonicator; Falc Instruments, Treviglio, Italy) for 30 min at 30 °C, and centrifuged at 6000 rpm at 4 °C for 15 min. The supernatants were filtered through Whatman No. 541 filter paper and stored at 18 °C until further analysis. Extracted samples were analyzed for condensed tannins, expressed as delphinidin equivalents (CT: g DE/kg DM) [8], using the butanol-HCl assay [25], and for total polyphenols, expressed as gallic acid equivalents (TP: g GAE/kg DM), using the Folin–Ciocalteau colorimetric method [26]. Absorbance of the samples was read at 550 nm for tannins and 725 nm for polyphenols using a HACH DR3900 spectrophotometer (Hach, Loveland, CO, USA).

### 2.4. In Vitro Fermentation Test

The sulla samples were incubated (1.0040 ± 0.0028 g) in 120 mL serum bottles with ruminal fluid from sheep (10 mL) at 39 °C under anaerobiosis [27]: Exp. 1 for 120 h in three replications for each substrate and Exp. 2 for 24 h in two replications for each substrate. For each experiment, serum bottles were incubated without substrate as control (blank) and two gas runs were performed on two consecutive days (in total 15 and 18 bottles including blanks for Exp. 1 and Exp. 2, respectively, for each gas run). The ruminal fluid was collected in an authorized slaughterhouse (EU, 2004) the day of the beginning of the trial from three adult sheep (Italian Merinizzata breed) raised on pasture. The collected material was placed in pre-heated thermoses and transported to the laboratory within 2 h of collection. All procedures involving animals were approved by the ethical committee for the care and use of animals at the University of Naples Federico II (Prot. 2019/0013729 of 8 February2019). The collected rumen fluid was pooled, mixed, and filtered through four layers of gauze by continuously flushing with CO_2_ for three hours and added (10 mL) to bottles containing the substrate, a buffer solution (75 mL), and a reducing agent (4 mL) composed according to Calabrò et al. [28]. The prepared bottles were then sealed with rubber stoppers and aluminum caps and incubated in a thermostat at 39 °C. The gas production was recorded at intervals of 2–24 h using a manual pressure transducer (Cole and Palmer Instrument Co, Vernon Hills, IL, USA) and the cumulative gas volume was related to the organic matter incubated (OMCV, mL/g). At the end of the incubation period, the fermentation liquid was analyzed for pH using a pH meter (ThermoOrion 720 A+, Fort Collins, CO, USA). The degradability of the organic matter (dOM, %) was determined as the difference in weight between the incubated organic matter and the residual matter after filtration through pre-weighted glass crucibles (porosity #2) and incineration in a muffle at 550 °C [29].

### 2.5. Final Fermentation Products

The fermentation liquor after 120 and 24 h of incubation was sampled and cooled to 4 °C to determine the content of volatile fatty acids (VFAs, mM/g). For this purpose, the samples were centrifuged at 12,000× *g* for 10 min at 4 °C (Universal centrifuge 32R, Hettich FurnTech Division DIY, Melle Neuenkirchen, Germany), and the supernatant (1.0 mL) was mixed with 1.0 mL of 0.06 mol oxalic acid. The different VFAs were evaluated using a gas chromatograph (Trace 1310, ThermoQuest Italia SpA, Rodano, Milano, Italy) equipped with a capillary column (30 m, 0.25 mm ID, 0.25 μm film thickness), using an external standard solution composed of acetic, propionic, butyric, iso-butyric, valeric, and iso-valeric acids. The percentage of branched-chain fatty acids (BCFAs, %) was calculated as (iso-butyric acid + iso-valeric acid)/total VFAs × 100.

### 2.6. Data Processing and Statistical Analysis

To study fermentation kinetics, gas production data recorded for 120 h for each bottle were fitted to the sigmoidal model (1) [30]:

(1) $$G\;=\;\frac{A}{\left(1+{\left(\frac{B}{t}\right)}^{c}\right)}$$

where G is the total gas produced at the end of incubation time (mL/g of incubated OM) at time t (h), A is the asymptotic gas production (mL/g), B is the time at which one-half of A is reached (h), and C is the curve switch. Then, maximum fermentation rate (Rmax, mL/h) and the time at which it occurs (Tmax, h) were calculated using Equations (2) and (3), respectively:

(2) $$Rmax=\frac{\left(A\times B^{C}\right)\times C\times {Tmax}^{\left(-C-1\right)}}{(\left(1+B^{C}\;\times \;{Tmax}^{-C}\right){)}^{2}}$$

(3) $$\mathrm{Tmax}=B\times {\left[\frac{C-1}{C+1}\right]}^{\frac{1}{C}}$$

The methane produced after 24 h of incubation (CH_4_, mL/g and % of total gas) was estimated from the volatile fatty acids [acetic (a), propionic (p), and butyric (b)] produced at 24 h, as proposed by Blummel et al. [31] in Formulas (4) and (5):

(4) $$mmol\;{CO}_{2ferm}=a/2+p/4+1.5b$$

(5) $$mmol\;{CH}_{4ferm}=a+2\times b-{CO}_{2}$$

The nutritive value of forages was estimated as metabolizable energy (ME, MJ/kg DM) using the Equation (6) proposed by Menke and Steingass [32]:

(6) $$ME=2.2+0.1357\times GP+0.0057\times CP+0.0002859\times CP^{2}$$

where CP is the content (g/kg DM) of the crude protein and GP is the gas obtained in vitro (mL/200 mg incubated DM) after 24 h of incubation. 

The in vitro fermentation parameters and final products were analyzed through a factorial model. 

In Exp. 1 a factorial model was applied considering the five substrates. One-way ANOVA was used to observe the differences between the substrates by the following formula:

$${Y_{ij}=\;\mu +\alpha_{i}+\varepsilon }_{ij}$$

where Y is the value of the dependent variable, μ is the general mean, α is the substrate effect (*i* = 1–5), and ε is the error effect.

In Exp. 2 a 2 × 4 factorial model was applied considering the cut period (1st and 2nd) and four different parts of the plant (whole plant vs. flowers vs. leaves vs. stems). Two-way ANOVA was used to observe the differences between the cut period and the different parts of the plant using the following formula:

$$Y_{ijk}=\;\mu +\alpha_{i}+\beta_{j}+\varepsilon_{ijk}$$

where Y is the value of the dependent variable, μ is the general mean, α is the cut effect (*i* = 1–2), β is the part plant effect (j = 1–4), and ε is the error effect.

The Shapiro–Wilk test was used in both experiments to assess the normality of the data distribution for the data (JMP, 2014).

Both analyses were conducted using the JMP^®^ statistical software package (Version 14 SW, SAS Institute Inc., Cary, NC, USA, 1989–2019) using Tukey’s HSD test for *p* < 0.05. The gas run replication was not significantly different and therefore in both statistical analyses it was not taken into consideration.

## 3. Results

### 3.1. Exp 1 Sulla Forages: Comparison of Different Preservation Systems

The data obtained from the chemical composition (Table 1) in all the forages tested in this trial highlight the high protein (CP: from 11.3 to 15.7% DM) and fair energy content (ME: from 6.54 to 10.3 MJ/kg DM), a moderate structural carbohydrate level (NDF: from 39.5 to 54.8% DM) with low lignin (ADL: from 5.31 to 8.16% DM), but a high amount of ash (from 10.1 to 14.4% DM). Among the samples analyzed, the ones with the best nutritional characteristics, in terms of content in protein (15.7 and 15.1% DM, in S1C_F and S1C_P, respectively), energy (10.3 and 9.64 MJ/kg DM, in S1C_F and S1C_P, respectively), fiber (40.1 and 39.5% DM in S1C_F and S1C_P, respectively), and lignin (6.03 and 5.31% DM in S1C_F and S1C_P, respectively) were the forages collected at the first cut both fresh and pelleted. Meanwhile, sulla preserved as hay (S_H) showed lower nutritional characteristics in terms of the structural carbohydrates level (54.8% DM). The yield of dehydrated forage, with 15% moisture, was approximately 25%, starting from fresh forage with a moisture content of 85%. 

**Figure 1:**
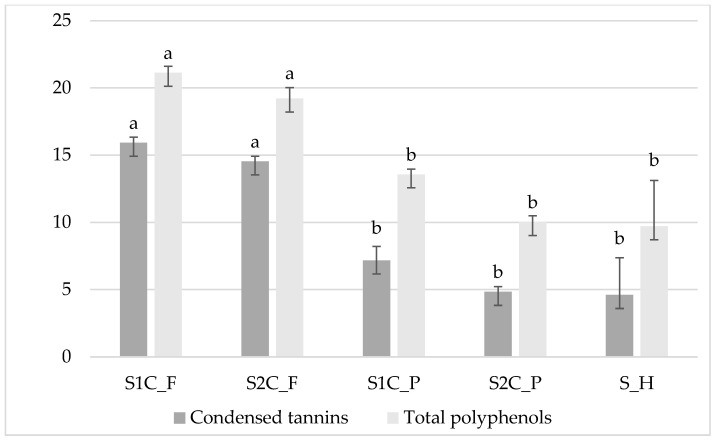
Total polyphenols (GAE g/kg DM) and condensed tannins (g/kg DM) of sulla forage of different preservation systems. S1C_F: sulla 1st cut fresh; S2C_F: sulla 2nd cut fresh; S1C_P: sulla 1st cut pelleted; S2C_P: sulla 2nd cut pelleted; S_H: sulla hay. Data expressed as Lsmean ± s.d. Condensed tannins: *p* value: 0.0001, SEM: 2.05; total polyphenols: *p* value: 0.0004, SEM: 1.31. Different letters indicate the statistical differences (*p* < 0.05) between sulla forage for condensed tannins and total polyphenols factors.

Relative to the plant’s secondary metabolite content (Figure 1), condensed tannins and total polyphenols decreased from first to second cuts in fresh and pelleted sulla (CT (g/kg DM): 15.9 and 14.5 in S1C_F and S2C_F and 7.17 and 4.83 in S1C_P and S2C_P; TP (GAE g/kg DM): 21.1 and 19.2 in S1C_F and S2C_F and 13.6 and 10.0 in S1C_P and S2C_P, respectively). 

**Figure 2:**
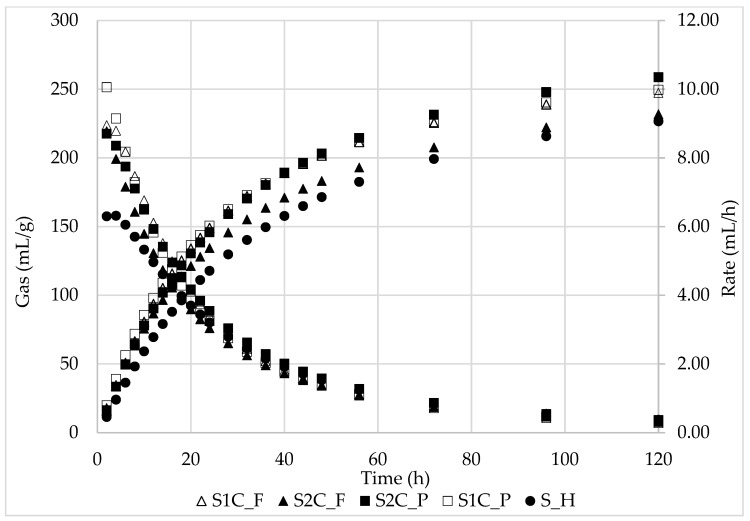
In vitro gas production and fermentation rate of sulla forage of different preservation systems. S1C_F: sulla 1st cut fresh; S2C_F: sulla 2nd cut fresh; S1C_P: sulla 1st cut pelleted; S2C_P: sulla 2nd cut pelleted; S_H: sulla hay.

Regarding the in vitro fermentation characteristics and final products after 120 h (Table 2 and Table 3), S2C_P and S1C_F exhibited the highest dOM (66.1 and 62.7%; *p* < 0.05); S1C_F, S1C_P and S2C_P showed the highest OMCV values (237, 246, 250 mL/g; *p* < 0.05). The same parameters were the lowest (*p* < 0.05) in S2C_F (dOM: 48.9%) and S_H (OMVC: 218 mL/g). Total volatile fatty acid (VFA) production was the highest (*p* < 0.05) in S1C_P, S_H, and S2C_P (87.3, 85.1, 83.6 mM/g) whereas the lowest (*p* < 0.05) was in S2C_F (64.1 mM/g). The branched-chain fatty acid percentage is related to the CP content, with the significant (*p* < 0.05) highest in S1C_P (9.11) and lowest in S_H (6.13). For all samples, the pH mean value was a little variable (6.34 ± 0.01). The in vitro fermentation kinetics (Table 2, Figure 2) indicate some differences in the first 24 h of incubation; in particular, gas production and fermentation rate are always lower for S_H than for the other samples. 

### 3.2. Exp 2 Comparison of Different Parts of the Sulla Plant

The percentage distribution of the various plant parts on the DM basis highlights that in the earlier cut there is a lower presence of flowers and a higher presence of leaves (S1C_F: 7.94 and 32.7%, in flowers and leaves, respectively) compared with the late cut (S2C_F: 25.9 and 18.8%, in flowers and leaves, respectively). In both cuts, stems are the most abundant plant parts (52.5 and 48.9% in S1C_F and S2C_F, respectively) and weeds represent a very small percentage (6.85 and 6.41%, in S1C_F and S2C_F, respectively). Regarding chemical composition (Table 4), on average the leaves in S1C_F and S2C_F show the highest nutritional value in terms of protein (26.3 and 27.3% DM) and lipid (4.15 and 4.46% DM) content, lower levels of structural carbohydrates (20.1 and 18.2% DM), followed by flowers and stems. This trend is consistent across the two cuts. The characteristics of in vitro fermentation, including methane estimation, after 24 h incubation and the content of secondary metabolites for different parts of the plant in the first and second cuts are shown in Table 5. Most of the parameters considered were statistically affected by cuts and plant parts, except gas production (OMCV). In the first cut (S1C_F), the values of leaves are significantly (*p* < 0.05) higher in organic matter degradability (57.31%), methane production (26.22 mL/g), and total volatile fatty acid production (43.29 mM/g) than flowers and stems; flowers have the lowest (*p* < 0.05) CH_4_ production (19.04 and 20.90 mL/g and % total gas); the secondary metabolite content is the highest (*p* < 0.05) in flowers and leaves (CT: 27.49, 26.60 g/kg DM; TP: 39.47, 36.50 GAE g/kg DM) and the lowest in stems (CT: 7.74 g/kg DM; TP: 9.37 GAE g/kg DM). In the second cut (S2C_F), flowers and leaves show the highest value (dOM: 53.32%; OMCV: 111.95 mL/g; CH_4_: 38.39 mL/g; VFA: 63.99 mM/g; CT 27.98 g/kg DM; *p* < 0.05) whereas stems show the lowest (OMCV: 90.17 mL/g; CT: 3.13 g/kg DM; TP: 5.64 GAE g/kg DM).

## 4. Discussion

Sulla is a Leguminosae forage considered of high quality for small ruminants, due to its rich protein content and the presence of beneficial secondary compounds. Previous studies reported that feeding fresh sulla forage improved lambs’ performance in terms of growth rate and carcass yield [33], sheep milk production and nutritional cheese quality [16], the antioxidant defenses of goats, and the oxidative stability of their milk [34]. These results were due to high DM and crude protein intake, and related to the favorable balance of protein, structural, and non-structural carbohydrates. Moreover, its moderate content of polyphenolic molecules, such as condensed tannins, enhances protein utilization by reducing protein degradation in the rumen, and results in a healthier lipid profile in dairy products by limiting the ruminal biohydrogenation of beneficial polyunsaturated fatty acids (PUFAs). Another distinguishing feature of sulla condensed tannins is their contribution to reducing gastrointestinal parasites and lowering the risk of meteorism [20]. 

Sulla is frequently compared to alfalfa (*Medicago sativa* L.) in terms of forage quality and productivity, although its overall biomass yield is lower (11.4 vs. 38.5 t DM/ha for sulla and alfalfa, respectively) [35]. Certainly, the harvest time is the factor that most influences nutritive value which rapidly decreases in sulla as the plant matures [2]. 

Overall, based on our results, sulla forage showed interesting nutritional characteristics proving it should be considered a valid legume in the Mediterranean area, comparable to alfalfa which, because of its high nutritional quality, yield, and adaptability, is one of the most widespread legume forages in the world. The sulla samples selected for this study correspond to the Avorio population (Landrace Avorio), which is highly appreciated by farmers in Sicily for its agronomic and nutritional characteristics, making its registration as a variety advisable. The differences that emerged in terms of nutritional characteristics (e.g., crude proteins, structural carbohydrates content) and in vitro fermentation characteristics moving from beginning to full flowering are due to changes in chemical composition and secondary metabolite content occurring during the growth of the plant. In particular, crude proteins are higher in young plants due to the presence of leaves whereas the structural carbohydrates are higher in mature plants to provide structural support for increased height and biomass. The secondary metabolite content is higher in young plants to focus on defense against pathogens. As reported in previous studies for sainfoin [36] and sulla [14,37] the rate of synthesis by the plant of these compounds increases during flowering and then slows down as the phenological stage advances towards late flowering. Condensed tannins in ruminants still have debated effects: it is reported that levels of less than 6.0% DM, such as those found in sulla, have a positive effect because they bind proteins at the ruminal level and promote their utilization in following traits, with improved performance [33]. However, in hot climates, higher contents of condensed tannins tend to be detrimental because they reduce protein availability in diets based on forages already deficient in protein [38]. In New Zealand, the better performance of animals grazing on sulla than on alfalfa has been attributed to the protective effects of condensed tannins on nematode infections [39]. In addition, milk from sheep grazing on sulla in bloom notwithstanding shown to have a lower content of conjugated linoleic acid, are richer in n-3 fatty acids, especially α-linolenic acid, and had a lower n-6:n-3 ratio than milk from sheep fortified with PEG which neutralizes the effect of condensed tannins in limiting the ruminal biohydrogenation of dietary PUFAs [40].

### 4.1. Sulla Forages: Comparison of Different Preservation Systems 

To ensure the availability of forage during periods of the year when it is not possible to use it fresh for grazing or cutting for feeding, various preservation systems are employed in agro-livestock farms. Haymaking is undoubtedly the most widespread technique, allowing legumes to perform better compared with ensiling. However, due to the loss of leaves, which contain the highest amount of nitrogenous nutrients, it is not always the best preservation method. Dehydrating the forage, which is then supplied as pellets, could be an effective preservation method for maintaining nutrients in sulla forage. Recent findings [16,17,41] conducted on small ruminants have shown that, overall, a diet based on dehydrated sulla pellets showed better results compared with hay and it was comparable to a diet based on fresh sulla in terms of milk production, casein levels in milk, and color, vitamin A, vitamin E and total PUFA content in cheese.

From the comparison of the different system proposed to preserve sulla forages (pelleted and hay), several interesting results and considerations emerge, both in terms of chemical composition, secondary metabolite content and in vitro fermentation characteristics and kinetics. Fresh forage harvested in both cuts confirmed the highest nutritional value because any preservation system (drying or processing system) reduces or modifies some nutrients: proteins are lost during the loss of leaves when moving hay windrows or denatured with the high temperature in pelleting; fiber increase due to the concentration effects and loss soluble components during the drying process. Fresh forage also showed higher lipid content than hay, presumably a PUFA, mainly α-linolenic acid, as reported by Gannuscio et al. [17], which will ensure better quality of milk and dairy products. Moreover, the total polyphenols content has a probable positive antioxidant effect, whereas the levels of total condensed tannins may only slightly interfere with ruminal microbial activities. The pelleted sulla maintained a high protein content and provided a fair level of NDF, especially in the 1st cut, without changing the lipid content in both cuts offering a good balance of nutrients essential for sheep health and productivity. These results are attributed to the pelleting system that minimized leaf loss, reduced oxidation and inactivated degrading enzymes. Sulla hay showed the highest content of structural carbohydrates and the lowest protein content, probably due to the haymaking process which during cellular respiration promotes the loss of soluble carbohydrates and during the turning of the forage mass favors the loss of nitrogen-rich leaves. During field drying, the content of condensed tannins and total polyphenols decreases because, due to their sensitivity to sunlight, heat and oxygen, their detectability and bioactivity is reduced. Concerning the in vitro fermentation characteristics (i.e., organic matter degradability, total gas and volatile fatty production), on average the pelleted forages showed the highest value, followed by fresh forage and then hay. Slower fermentation kinetics (i.e., gas production and fermentation rate) emerged in hay, followed by both cuts of fresh forage and then pelleted. In contrast Calabrò et al. [28] in a trial performed using *Avena sativa* L., found higher total gas production for the hay than for fresh plant. Overall, the effects on in vitro outcome could be mainly related to the chemical composition (NDF and crude protein contents) but also to the content of secondary metabolites in legume forage which, by binding to proteins, negatively interfere with their availability by modifying the rumen bacterial activity and therefore fermentation. The pH mean value after incubation indicated that the in vitro fermentation ensures a favorable environment for cellulolytic bacterial activity [42]. Trials performed in vivo showed that sulla is quite digestible, reporting values up to 75.0% for OM and greater than 70.0% for ruminal degradability of protein [2,43]. Similarly, Pipi [41] found values of in vivo OM and protein digestibility equal to 75% and 68% for fresh sulla forage, 77% and 74% for dehydrated and pelleted sulla, and 69% and 68% for hay, respectively.

### 4.2. Nutritional Characteristics of Different Parts of the Sulla Plant

Our research investigates the influence of nutrient and secondary metabolite content in three different parts of fresh sulla forage on in vitro fermentation characteristics and the estimation of methane production. Scientific studies evaluating the nutritional value of forage botanical parts have also been conducted by other authors, since the grazing behavior of ruminants is often selective towards specific plant organs (e.g., flowers, leaves, stems). Therefore, their concentrations in nutritive and non-nutritive compounds can differently influence rumen fermentations and the metabolism of microorganisms. Specifically, as reported, sulla leaves, stems, and flowers are rich in condensed tannins, with levels ranging from 0.8 to 5.0% DM [11] and from 3.0 to 12.0% DM [4], with variations due to environmental and climatic conditions, growth stage, and plant genotype. However, higher levels of condensed tannins are detected in leaves and flowers [8,11,37], in line with our results.

Sulla is a highly palatable forage for ruminants, which graze and prefer it mixed in with cereal pastures, especially before flowering, when its quality peaks; however, as flowering progresses, the stems become more fibrous and less palatable [44].

Moreover, Woodward et al. [45] reported that sulla tannins may contribute to reducing methane emissions in ruminants. In a study conducted in New Zealand, cows grazing on sulla produced less methane per unit of DM ingested (19.5 vs. 24.6 g CH_4_/kg DM) and per liter of milk produced (243.3 vs. 327.8 g CH_4_/kg milk DM) than cows fed perennial ryegrass, both due to the higher nutritional value of sulla, in terms of protein to fiber ratio, energy, and the presence of condensed tannins; similar results were obtained by Pipi [41] estimating methane production (g/kg milk) of ewes fed fresh forage of sulla or barley. In our study, the results of methane production related to secondary metabolites did not show a clear trend. However, in the first cut, the flowers, related to the highest presence of secondary metabolites that also influenced dOM and VFAs, showed the lowest CH_4_ production due to their ability to inhibit the activity of methanogens. In the second cut, the effect on CH_4_ production is likely linked to the higher content of structural carbohydrates—resulting from a more advanced phenological stage—which promotes greater methane formation. This is also confirmed by the higher amount of total VFAs produced [36].

## 5. Conclusions

The results of the investigation obtained with the in vitro cumulative gas production technique showed that sulla forage harvested at the beginning of flowering and preserved as dehydrated pellet has interesting nutritional characteristics (CP: 15.1%DM, ME. 9.64 MJ/kg DM, TP: 13.57 GAE g/kg DM, dOM: 55.7%) that show it should be considered a valid legume forage resource of high-quality value. Pelleting, compared to hay, proved to be an effective preservation method for maintaining nutrients in sulla forage due to the reduced loss of leaves and lower effect of some factors (i.e., light, temperature, and oxygen). Considering that small ruminants prefer higher quality forages and have less need for long fiber, as they are selective and have physical limitations, pelleted forage seems to be a useful alternative to hay to enhance animals’ performance and the quality standard of their products. Notwithstanding these advantages, its practical use in livestock farms could be conditioned by the cost of the system. As hypothesized, the secondary metabolites influenced rumen microbial activity. Considering the different parts of the plant studied, total polyphenols and condensed tannins influenced the in vitro fermentations with beneficial effects on the environment (e.g., methane reduction). The findings of this study warrant further investigation, particularly in view of their potential environmental implications. Sulla (*Hedysarum coronarium* L.), as a leguminous species well-adapted to arid and semi-arid environments, not only enhances soil nitrogen content through biological nitrogen fixation but also contributes to methane mitigation due to the presence of bioactive secondary metabolites. In perspective, it is expected that the findings of this study should contribute to the development of sustainable dehydration techniques to produce pellets of sulla forage and induce future investigations focusing on the bioavailability of polyphenols and their impact on oxidative stability and the organoleptic, nutritional, and health quality of meat and dairy products.

## Figures and Tables

**Table 1:** Chemical composition of sulla forage of different preservation systems.

Sample	DM	CP	EE	Ash	NDF	ADF	ADL	NSC	ME
	%	% DM							MJ/kg DM
S1C_F	16.0	15.7	2.40	10.6	40.1	35.7	6.03	31.1	10.3
S2C_F	19.5	14.7	2.55	10.1	46.4	43.4	8.16	24.7	9.34
S1C_P	86.6	15.1	2.32	14.4	39.5	32.0	5.31	28.7	9.64
S2C_P	87.8	11.3	2.65	12.4	52.5	41.8	7.17	21.2	6.54
S_H	90.2	11.8	1.39	11.5	54.8	42.9	7.88	20.5	6.91

S1C_F: sulla 1st cut fresh; S2C_F: sulla 2nd cut fresh; S1C_P: sulla 1st cut pelleted; S2C_P: sulla 2nd cut pelleted; S_H: sulla hay; DM: dry matter; CP: crude protein; EE: ether extract; NDF: neutral detergent fiber; ADF: acid detergent fiber; ADL: acid detergent lignin; non-structural carbohydrate (NSC = 100 − (%NDF + %CP + %EE + %Ash)); ME: metabolizable energy.

**Table 2:** In vitro fermentation of sulla forage of different preservation systems.

Sample	dOM	OMCV	Tmax	Rmax
	%	mL/g	h	mL/h
S1C_F	62.7 ^a^	237 ^ab^	1.46 ^c^	9.84 ^a^
S2C_F	48.9 ^c^	222 ^bc^	0.27 ^d^	10.0 ^a^
S1C_P	55.7 ^b^	246 ^a^	1.48 ^c^	9.86 ^a^
S2C_P	66.1 ^a^	250 ^a^	2.51 ^b^	8.44 ^a^
S_H	52.0 ^bc^	218 ^c^	3.69 ^a^	6.18 ^b^
*p* value	0.0002	0.0006	4.56 × 10^6^	0.0001
SEM	0.91	0.89	0.87	0.55

S1C_F: sulla 1st cut fresh; S2C_F: sulla 2nd cut fresh; S1C_P: sulla 1st cut pelleted; S2C_P: sulla 2nd cut pelleted; S_H: sulla hay; dOM: organic matter degradability, OMCV: cumulative volume of gas related to incubated organic matter; Tmax: time at which maximum rate was reached, Rmax: maximum fermentation rate. SEM: standard error of mean. Along the column, different letters indicate the statistical differences (*p* < 0.05).

**Table 3:** Final fermentation products of sulla forage of different preservation systems.

Sample	Ace	Prop	Iso-But	But	Iso-Val	Val	VFA	BCFA	A/P
	mM/g							%	
S1C_F	45.8 ^c^	14.8 ^b^	1.33 ^b^	6.60 ^b^	2.38 ^bc^	2.09	73.0 ^b^	6.50 ^bc^	3.96 ^bc^
S2C_F	40.4 ^d^	14.4 ^b^	1.31 ^b^	4.03 ^c^	2.37 ^c^	1.60	64.1 ^c^	7.44 ^b^	3.51 ^d^
S1C_P	53.4 ^a^	16.3 ^b^	1.59 ^b^	9.17 ^a^	3.78 ^a^	2.32	87.3 ^a^	9.11 ^a^	4.32
S2C_P	50.2 ^b^	19.7 ^a^	2.25 ^a^	7.31 ^b^	2.80 ^b^	1.92	83.6 ^a^	7.24 ^b^	3.51 ^d^
S_H	53.2 ^a^	16.0 ^b^	1.45 ^b^	8.84 ^a^	2.68 ^bc^	2.90	85.1 ^a^	6.13 ^c^	4.20 ^ab^
*p* value	1.88 × 10^8^	4.31 × 10^5^	5.14 × 10^6^	4.85 × 10^8^	4.70 × 10^6^	2.62 × 10^8^	1.07 × 10^5^	1.27 × 10^7^	7.97 × 10^5^
SEM	0.72	0.46	0.28	0.70	0.32	0.32	0.99	0.40	0.18

S1C_F: sulla 1st cut fresh; S2C_F: sulla 2nd cut fresh; S1C_P: sulla 1st cut pelleted; S2C_P: sulla 2nd cut pelleted; S_H: sulla hay. VFA: total volatile fatty acid; Ace: acetate; Pro: propionate; Iso-But: iso-butyrate; But: butyrate; Iso-Val: iso-valerate; Val: valerate; BCFA: branched-chain fatty acid; A/P: acetate to propionate ratio. SEM: standard error of mean. Along the column, different letters indicate the statistical differences (*p* < 0.05).

**Table 4:** Chemical composition in different parts of the fresh sulla.

Sample	DM	CP	EE	Ash	NDF	ADF	ADL	NSC	ME
	%	% DM							MJ/kg DM
	S1C_F								
Whole plant	16.0	15.7	2.40	10.8	40.1	35.7	6.03	32.1	10.3
Flowers	18.0	19.7	2.90	7.85	32.9	27.2	5.25	36.6	14.5
Leaves	18.9	26.3	4.15	13.0	20.1	19.4	3.36	36.4	23.6
Stems	13.6	8.61	1.21	9.25	53.5	46.6	7.77	27.4	4.88
	S2C_F								
Whole plant	19.5	14.7	2.55	10.1	46.4	43.4	8.16	24.7	9.34
Flowers	21.8	20.6	3.37	8.20	36.9	32.3	5.58	30.9	15.6
Leaves	17.9	27.3	4.46	14.6	18.2	17.8	4.00	35.4	25.2
Stems	18.6	6.51	1.30	8.63	63.3	59.8	11.1	20.3	3.86

S1C_F: sulla 1st cut fresh; S2C_F: sulla 2nd cut fresh. DM: dry matter, CP: crude protein, EE: ether extract, NDF: neutral detergent fiber, ADF: acid detergent fiber, ADL: acid detergent lignin, NSC: non-structural carbohydrate (NSC = 100 − (%NDF + %CP + %EE + %Ash)); ME: metabolizable energy.

**Table 5:** In vitro fermentation characteristics at 24 h of incubation and secondary metabolite content in different parts of the fresh sulla.

Sample	dOM	OMCV	CH_4_	CH_4_	VFA	CT	TP
	%	mL/g	mL/g	% gas tot	mM/g	g/kg DM	GAE g/kg DM
	S1C_F						
Whole plant	54.65 ^a^	98.64	25.47 ^a^	25.83 ^a^	42.98 ^a^	15.92 ^b^	21.12 ^b^
Flowers	43.02 ^b^	91.31	19.04 ^b^	20.90 ^b^	34.95 ^b^	27.49 ^a^	39.47 ^a^
Leaves	57.31 ^a^	100.3	26.22 ^a^	26.14 ^a^	43.29 ^a^	26.60 ^a^	36.50 ^a^
Stems	37.09 ^c^	87.83	24.75 ^a^	28.27 ^a^	41.99 ^a^	7.74 ^c^	9.37 ^c^
	S2 C_F						
Whole plant	38.91 ^b^	100.11	17.23 ^b^	17.22 ^b^	29.86 ^c^	14.53 ^b^	19.21 ^b^
Flowers	53.32 ^a^	111.95	38.39 ^a^	33.65 ^a^	63.99 ^a^	27.98 ^a^	32.46 ^a^
Leaves	47.61 ^ab^	90.20	32.97 ^a^	36.55 ^a^	53.89 ^ab^	26.94 ^a^	38.16 ^a^
Stems	39.97 ^b^	90.17	26.23 ^ab^	29.07 ^ab^	43.42 ^b^	3.13 ^c^	5.64 ^c^
	P significance						
Effect cut	0.17362	0.3670	0.1080	0.0327	0.1582	0.0026	0.0227
Effect plant part	0.00839	0.1690	0.2000	0.0003	0.2221	5.41 × 10^1^	6.1 × 10^8^
Effect interaction	0.00778	0.1050	0.0370	0.0395	0.0716	0.0010	0.0727
SEM	1.09	0.95	1.52	1.26	1.90	1.82	2.09

S1C_F: sulla 1st cut fresh; S2C_F: sulla 2nd cut fresh. dOM: organic matter degradability; OMCV: cumulative volume of gas related to incubated organic matter; CH_4_: methane production estimated from VFA; VFA: total volatile fatty acid; CT: condensed tannins; TP: total polyphenols. SEM: standard error of mean. Along the column, for each cut, different letters indicate the statistical differences (*p* < 0.05).

## Data Availability

The original contributions presented in this study are included in the article. Further inquiries can be directed to the corresponding author.

## Abbreviations

The following abbreviations are used in this manuscript:

DM	dry matter
CP	crude protein
EE	ether extract
NDF	neutral detergent fiber
ADF	acid detergent fiber
ADL	acid detergent lignin
NSC	non-structural carbohydrate
ME	metabolizable energy
dOM	organic matter degradability
OMCV	organic matter cumulative volume
Tmax	time at which maximum rate was reached
Rmax	maximum fermentation rate
MSE	mean square error
VFAs	total volatile fatty acids
Ace	acetate
Pro	propionate
Iso-but	iso-butyrate
But	butyrate
Iso-Val	iso-valerate
Val	valerate
BCFA	Branched-chain fatty acid
A/P	acetate to propionate ratio
CT	condensed tannins
TP	total polyphenols
PUFAs	polyunsaturated fatty acids
NS	not significant
S1C_F	sulla 1st cut fresh
S2C_F	sulla 2nd cut fresh
S1C_P	sulla 1st cut pelleted
S2C_P	sulla 2nd cut pelleted
S_H	sulla hay
OM	organic matter
A	asymptotic gas production
B	time at which one-half of A is reached
C	is the curve switch
a	acetic
p	propionic
b	butyric
GP	gas production obtained in vitro after 24 h of incubation
